# Toluidine blue: rapid and simple malaria parasite screening and species identification

**DOI:** 10.11604/pamj.2017.28.27.12488

**Published:** 2017-09-13

**Authors:** Rupali Awale, Ratnaprabha Maji, Parag Patil, Raghavendra Lingiah, Ashok Kumar Mukhopadhyay, Subhadra Sharma

**Affiliations:** 1 All India Institute of Medical Sciences, New Delhi, India

**Keywords:** Leishman stain, malaria, screening, toluidine blue stain

## Abstract

Malaria, a febrile illness mostly confined to the tropical countries is transmitted by bite of infected female Anopheles mosquito. In 2015 alone, 88% of the malaria burden and 90% deaths due to malaria were confined to the African and Asian countries. Although number of tests are available for rapid diagnosis and screening for malaria, peripheral blood smear examination remains the gold standard. Leishman stain is recommended by WHO however herein we evaluate one of the alternative methods of staining which is simple and rapid. Fifty patients attending the various outpatient departments of the tertiary care hospital with fever and suspected to have malaria were selected. Two thin-air dried smears prepared from the peripheral venous blood from these subjects were stained by Leishman and Toluidine blue method. The findings of the slides by two independent qualified professionals were noted and the results were analyzed. A total of 14% (7/50) cases were diagnosed to have malaria. All the malaria cases which were positive in Leishman stain were also detected in Toluidine blue stain. Malarial parasites were clearly visible against the homogenously light green background in Toluidine blue. The detection of malarial parasite by Toluidine blue was quick, easy and confirmative. Toluidine blue stained peripheral blood smear allows for easy identification and speciation of malarial parasite at low magnification and in shorter period of time.

## Introduction

Malaria is a febrile illness, often life threatening which is transmitted to people through the bite of infected female Anopheles mosquito. Worldwide in the year 2015 an estimated 21,2000,000 cases of malaria were seen while 429,000 deaths had occurred [1, 2]. African regions still continue to bear the brunt of global burden of malaria due to a combination of factors like high vector population, favorable weather for transmission throughout the year and more number of infection by plasmodium falciparum which causes high morbidity and mortality. In 2015 itself, 88% of the global cases and 90% of global deaths due to malaria occurred in Africa. India is also one of the countries with high malaria transmission rate [1, 2]. Till date various methods are available for screening and diagnosis of malaria, they range from rapid detection of malaria antigens to molecular techniques. Examination of the blood smear under microscope is one of the earliest described and economic methods of all the above. Both thick and thin smears can be used. For preparing thick smears expertise is required in making the blood smear, dehemoglobinisation, drying and then staining. In a study the author concluded that thick smears when stained with Giemsa aid in the detection of malarial parasite especially with low density. But it requires time and experience so becomes impractical with high sample loads. Separate stains were required for both thick and thin smears, in the thick smears where parasite identification is the focus Giemsa stain is superior as compared to Leishman [3, 4].

Similarly, acridine orange fluorescent microscopy can be used in the same setting but needs expertise and is expensive [3]. Few authors have demonstrated that Giemsa stained thick films when used in study of parasite can significantly underestimate parasite density and also has reduced sensitivity [5–8]. In Quantitative buffy coat the parasitized red blood cells can be directly observed in the ultraviolet illuminated tubes but is an expensive matter [8]. While the commercially available acridine orange stained capillary tube preparation cannot identify the species nor detect the extra-erythrocytic parasites [9]. Few authors recommend acridine orange for screening if facilities are available [10]. Malaria Antigen detection is a rapid diagnostic test to detect evidence of malaria parasite in blood especially in areas with limited access to good quality microscopy services, but the cost is high as compared to microscopy [1, 2]. Inspite of all the advances so far, identification of the malarial parasite in the peripheral smear remains the gold standard for diagnosing malaria [11]. Various methods are in use to assess the presence of parasite in the field area but every test has its limitation so there is a need of a new staining technique. Toluidine blue has high affinity for acidic tissue components as it is a basic thiazine metachromatic dye. Since its discovery in 1856 by William Henry Perkin it has been used extensively for various medical applications like staining of connective tissue mucins, mast cell granules, amyloid microorganism like corynaebacterium diphtheria, helicobacter and mycobacterium and even malignant oral cells [12]. Toulidine blue has been used for the evaluation of the malarial parasite in smears previously [11, 13]. However, inspite of all advances to our knowledge toluidine blue stain has not been utilized in a large scale for malaria diagnosis. In this study we intend to evaluate its utility and efficacy in screening and species identification of parasites causing malaria at an institutional setup.

## Methods

The study was conducted over a period of one month at the All India Institute of Medical Sciences, India. Fifty patients attending the various outpatient departments of the tertiary care hospital with fever and suspected to have malaria were selected. Two peripheral blood thin smears were made from the blood collected in EDTA vacutainer within four hours of collection. After air drying one slide was subjected to staining by Leishman stain while the other by Toluidine blue stain. Leishman stain was prepared in a flask by mixing 1.5 grams of Leishman powder with 500 ml methanol followed by warming the mixture to 37 degree Celsius for overnight with occasional shaking. After cooling the stain was filtered and used. Leishman staining was done on one of the slides. The air dried smear was flooded with Leishman stain, after 2 minutes double quantity of distilled water was added and thoroughly mixed with the help of a pipette and kept for 8 minutes followed by washing in a stream of tap water. The back side of the slide was wiped clean with dry cotton and set upright for drying for 5 minutes [14]. The Leishman stained smear was then subjected to microscopic examination. Toluidine blue stain was prepared in a flask by mixing 5 grams of Toluidine blue stain with 100 ml of 95% alcohol and 400 ml of distilled water followed by keeping the mixture at room temperature overnight. Thereafter the stain was filtered and used. Toluidine blue staining was done on one of the slides. The air dried smear was flooded with Toluidine blue stain for 1 minute followed by washing in a stream of deionized water. The back side of the slide was wiped clean with dry cotton and set upright for drying for 5 minutes. The Leishman stained smear was then subjected to microscopic examination. The slides were reported by two different qualified lab professionals in a blind folded manner. The findings were noted and data was analyzed.

## Results

**Figure 1:**
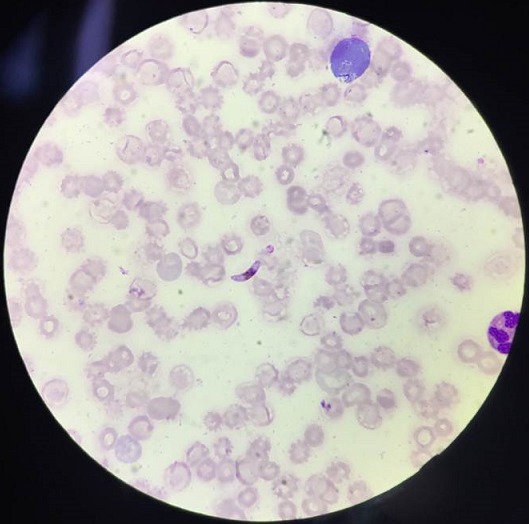
Photomicrograph of peripheral blood smear showing plasmodium falciparum (Leishman stain 1000X)

**Figure 2:**
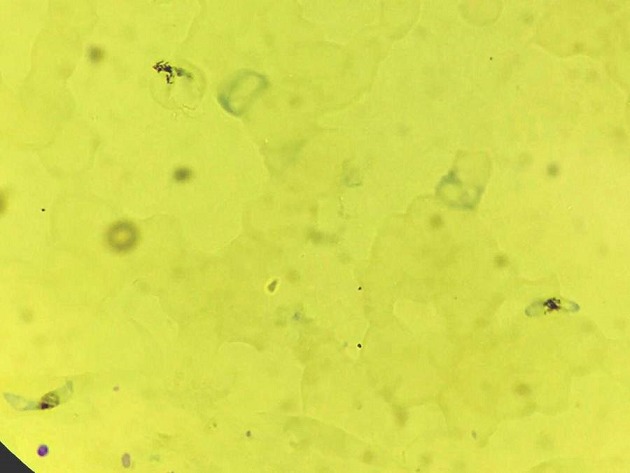
Photomicrograph of peripheral blood smear showing plasmodium falciparum (Toluidine blue stain 1000X)

**Figure 3:**
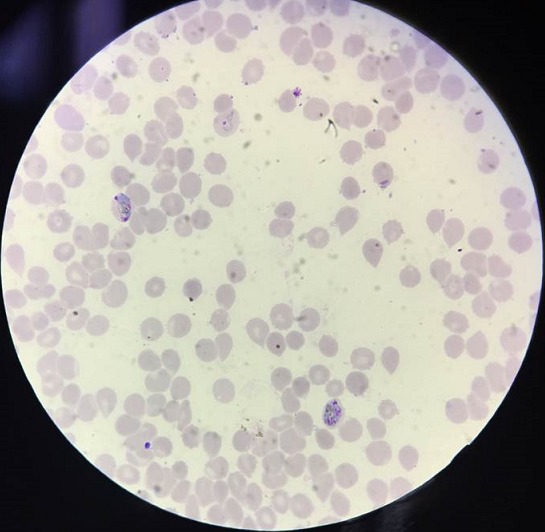
Photomicrograph of peripheral blood smear showing plasmodium vivax (Leishman stain 1000X)

**Figure 4:**
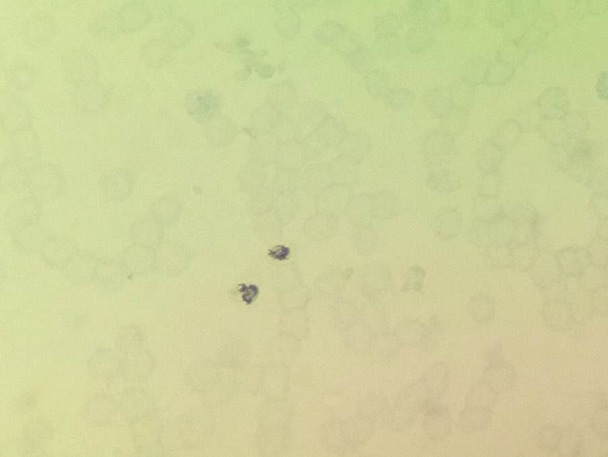
Photomicrograph of peripheral blood smear showing plasmodium vivax (Toluidine blue stain 1000X)

The patients belonged to all age groups from 2 months to 70 years with a mean of 27.4 years. Maximum number of cases (28%) was seen in the 2^nd^ decade. The age wise distribution of cases is as shown in Table 1. Male to female ratio was 1.2:1. Out of the fifty cases, only seven which is about 14% were diagnosed positive for malaria by examining Leishman stained smears which is the gold standard recommended method. All the slides reported positive for malaria in Leishman stain were also found to be detected by toluidine staining method. In the toluidine blue stained slides the parasite was distinctly visible with dark blue staining of the pigment while the background was homogenously light green stained. (Figure 1, Figure 2, Figure 3, Figure 4) In the Leishman stained smears red blood cells, white blood cells and platelets were stained. The red blood cells were stained brownish while the parasitized red blood cells showed the nucleus of the parasite as blue and dark pinkish granules. The parasite index ranged from 0.003 to 0.04; mean 0.02. The time duration along with staining pattern is as shown in Table 2. In microscopic examination of Toluidine blue stained smears the parasite is visible even in low power lens of 200X magnification as dark bluish green dots amidst a light green background thus serving the purpose of screening while the fine morphological details of the parasite are clearly visible in 400X magnification. While in the Leishman stained smears the parasite was visible only at 400X magnification while the fine morphological details were identified at 1000X magnification only.

**Table 1:** Age wise distribution of cases

Age (years)	All cases		Malaria positive cases	
	Number	%	Number	%
0-10	6	12	-	
11-20	14	28	2	28.5
21-30	10	20	1	14.2
31-40	9	18	1	14.2
41-50	7	14	2	28.5
51-60	2	4	1	14.2
61-70	2	4	-	-
Total	50	100	7	100

**Table 2:** Comparison of the two staining methods

Comparison of	Leishman stain	Toluidine blue stain
Mean duration of staining	15-18 minutes	6-8 minutes
Average screening time per slide	10-15 minutes	5-8 minutes
Staining pattern	The cellular blood components – red blood cells, white blood cells and platelets were stained.	The cellular blood components not stained and appear as homogenous light green stained like ghost of host cells.
	In Pl. falciparum- Crescentic shaped gametocyte with light blue nucleus, Trophozoite as single delicate, small blue ring.	In Pl. falciparum- Crescentic shaped gametocyte outline stained light blue while, Trophozoite as single delicate, small dark greenish black ring.
	In Pl. vivax- large and spherical gametocyte with light blue nucleus. Trophozoite- multiple thick rings with red chromatin at thinner part of ring	In Pl.Vivax- Large and spherical gametocyte with prominent dark blue nucleus. Trophozoite- multiple thick dark greenish black rings.

### Discussion

Malaria can be detected by different staining procedures some authors have reported the need for separate stains for thick and thin smears. Giemsa stain is the preferred method for thick smear where the focus is to easily detect the parasite of parasite and to increase the sensitivity, while Leishman stain is recommended for thin smears for identification of species. However, it is necessary to maintain the pH of 7.2 when using either of the two methods of staining. Toluidine blue stain which is used in this study showed comparable results as with Leishman stain in speciation and easy detection of parasites like Giemsa stain. Hence this single stain can be used on thin smears for both purposes. Moreover maintenance of optimal pH is not required making it useful for implementation in resource limited and field setting [14]. Also, as parasites can be identified even at low power objective, large areas of smear can be screened in short period of time, this is especially useful in cases of low parasite load as demonstrated in the present study [13]. Few studies have reported a higher detection rate (upto 5% higher) as compared to conventional Leishman staining method along with more specificity and accuracy [11, 13]. However in our study both methods showed equal sensitivity and specificity. Other quick staining methods like Fields stain have also been used for demonstration of malarial parasites but it lacks sensitivity as shown in one of the studies where Field's stain missed 65.4% of samples positive with Leishman stain [15]. Toluidine blue in spite of being quick stain did not miss any malaria positive cases. Antigen detection kits are rapid and can be used in case of emergency where there is high clinical suspicion and facilities for detailed microscopy are not available. Although it is rapid, it is also expensive ,there is need to assure the quality with lot to lot shipments and patients show positive results for weeks even after the successful treatment which can be misleading. Toluidine blue method requires no expertise in staining and microscopy, as only the parasites are stained it can be used as a rapid and easy alternative to other staining methods [1, 2, 14, 15].

## Conclusion

Toluidine blue stained peripheral blood smear thus allows for easy identification and species categorization of malarial parasite within shorter period of time and at a very low cost. Thus it can be superior in comparison to other stains for rapid detection of malarial parasite. The limitation of the study is that it is done in limited number of samples, so a large scale study is required for establishing the toluidine blue stain.

### What is known about this topic

- Toluidine blue is a metachromatic dye used to stain structures like staining of connective tissue mucins, mast cell granules, amyloid microorganism like corynaebacterium diphtheria, helicobacter and mycobacterium and even malignant oral cells;
- Malaria screening and diagnosis is done by various methods like staining of thick blood films by Giemsa stain for screening and staining of thin blood films by Leishmna stain for species identification;
- The known methods are time consuming and also expensive.

### What this study adds

- Screening and species identification can be done in the toluidine blue stained smear as only the malarial parasite is stained in the ghost of host cells;
- The toluidine blue method is cheap, expertise not required, less turnaround time and without compromise on the quality;
- The various species of malaria along with the various stages like gametocyte, ring forms are all visible in their fine morphological details.

## Competing interests

The authors declares no competing interests.
